# The interaction between endogenous 30S ribosomal subunit protein S11 and *Cucumber mosaic virus* LS2b protein affects viral replication, infection and gene silencing suppressor activity

**DOI:** 10.1371/journal.pone.0182459

**Published:** 2017-08-14

**Authors:** Ruilin Wang, Zhiyou Du, Zhenqing Bai, Zongsuo Liang

**Affiliations:** 1 Northwest Agriculture and Forestry University, College of Life Science, Yangling, Shaanxi, China; 2 Xian Mision Bio-Tech, Xian, Shaanxi, China; 3 Zhejiang Sci-Tech University, College of Life Science, Hangzhou, Zhejiang, China; National University of Singapore, SINGAPORE

## Abstract

*Cucumber mosaic virus* (CMV) is a model virus for plant–virus protein interaction and mechanism research because of its wide distribution, high-level of replication and simple genome structure. The 2b protein is a multifunctional protein encoded by CMV that suppresses RNA silencing-based antiviral defense and contributes to CMV virulence in host plants. In this report, 12 host proteins were identified as CMV LS2b binding partners using the yeast two-hybrid screen system from the *Arabidopsis thaliana* cDNA library. Among the host proteins, 30S ribosomal subunit protein S11 (RPS11) was selected for further studies. The interaction between LS2b and full-length RPS11 was confirmed using the yeast two-hybrid system. Bimolecular fluorescence complementation (BIFC) assays observed by confocal laser microscopy and Glutathione S-transferase (GST) pull-down assays were used to verify the interaction between endogenous NbRPS11 and viral CMVLS2b both *in vivo* and *in vitro*. TRV-based gene silencing vector was used to knockdown NbRPS11 transcription, and immunoblot analysis revealed a decline in infectious viral RNA replication and a decrease in CMV infection in RPS11 down-regulated *Nicotiana* *benthamiana* plants. Thus, the knockdown of RPS11 likely inhibited CMV replication and accumulation. The gene silencing suppressor activity of CMV2b protein was reduced by the RPS11 knockdown. This study demonstrated that the function of viral LS2b protein was remarkably affected by the interaction with host RPS11 protein.

## Introduction

*Cucumber mosaic virus* (CMV) has long been studied as an important plant virus model for understanding plant–virus interactions because of its broad host range and typical viral genome. CMV infects over 1200 species of plants, including many significant economic crops [1]. The CMV genome consists of three single-stranded positive-sense RNAs, namely, RNA1, RNA2 and RNA3 (sorted by decreasing size), which encode five open-reading frames (ORFs): 1a protein by RNA1, 2a and 2b proteins by RNA2, and movement protein (MP) and capsid protein (CP) by RNA3. The subgenomic RNA4 and RNA4A encode ORFs of CP and 2b protein respectively. The 110 amino acid multifunctional 2b protein from subgroup I is among the first viral proteins identified as RNA silencing suppressors (VSR) [1]. The 2b protein was initially identified as a virulence factor [2] in a strain of CMV that lacked the 2b gene, which resulted in an asymptomatic infection. Several studies eventually confirmed the biological function of 2b protein as a determinant of pathogenicity. Particularly, the 2b protein influences the dynamics of virion movement [3] and inhibits host defense mechanisms [4]. CMV 2b protein also binds small RNA duplexes and longer dsRNAs [5].

Two tonoplast intrinsic proteins from *Arabidopsis thaliana* interact *in vitro* with protein CMV 1a to colocalize in transfected protoplasts; this phenomenon suggests that these proteins participate in protein 1a anchoring or virus replication [6]. Movement of virions through plasmodesmata was associated with the interaction between the central part of MP and F-actin filaments [7]. Interactions between Tsip1 from tobacco and 1a and 2a proteins regulate virus replication [8]. Another protein from tobacco, Tcoi1, interacts with protein 1a, and systemic accumulation of the virus increased in plants expressing Tcoi1 [6]. Thus, host protein interactions with viral proteins remarkably affect viral function. Plant–virus interactions are difficult to understand because only a few host proteins that interact with CMV proteins have been identified. Further studies are required to understand the biomechanisms of host protein interaction and the influences on virus infection. Mapping the interaction partners of CMV 2b protein provides important clues to understand how the viral protein mediates normal biological processes in host cells. CMV 2b protein binds small RNA duplexes and longer dsRNAs to a lesser extent *in vivo* and *in vitro* [5].

CMV strains are generally characterized and phylogenetically divided into three subgroups (i.e., IA, IB, and II), which share at least 70% conservative nucleotide identity[1]. Determinants for virulence have been mapped on the 2b sequence of subgroup IA strain Fny-CMV [9]. Each strain of 2b protein shows differences of subcellular localization and function. The primary difference in virulence of all CMV strains is mediated by the differences in their respective 2b proteins. The members of subgroup II are generally milder than subgroup I strains based on their virulence; LS-CMV 2b was selected from subgroup II strains to interact with the *Arabidopsis* cDNA library.

Among all the host proteins identified as CMV LS2b binding partners by the yeast two-hybrid screen system, RPS11 was selected for further studies because 30S ribosomal subunit scored the largest proportion of all positive clones. RPS11 has a typical fold of α-helix packed against β-sheet and a long N-terminal tail for its secondary structure in domains. Given that 30S ribosomal protein is a key to protein synthesis [10], investigating the interaction between endogenous RPS11 and viral protein can elucidate the effect of host protein on viral infection. Protein–protein interactions play essential roles in many endogenous- and exogenous-related biological phenomena. Pull-down assays are an effective initial screening assay *in vitro* to identify and confirm the existence of protein-protein interactions. In living cells, the bimolecular fluorescence complementation (BiFC) assay is widely used to detect protein–protein interactions. However, further research is required because only a few endogenous proteins that interact with viral proteins have been identified. In the current work, we performed pull-down and BiFC assays to verify the interaction between RPS11 and CMVLS2b proteins. Virus-induced gene silencing (VIGS) has been developed as an effective technique in post-transcriptional gene silencing (PTGS) in research. A gene silencing approach with *N*. *benthamiana* as the host was used to investigate the role of RPS11 in CMV replication and accumulation. We cloned RPS11 from *N*. *benthamiana* inserted into a tobacco rattle virus (TRV)-based gene silencing vector. The CMV RNA accumulation in plants was tested with RPS11 silencing to down-regulate at day 7. Results suggested that the change in RPS11 expression affected the CMV infectious viral RNA replication, viron accumulation, and gene silencing suppression of CMV 2b protein.

## Results

### Identification of CMV LS2b-interacting partners using the yeast two-hybrid screen system

Considering the importance of the LS2b protein in CMV-host interaction, our goal was to understand its role in virus accumulation. A yeast two-hybrid screen of the *A*. *thaliana* cDNA library was performed to identify the interacting partners of the host. LS2b bait was first tested; autoactivation and toxicity results showed that the bait LS2b protein did not autonomously activate the reporter genes in the absence of prey protein (S1 Fig). Based on normal growth progress on both solid and liquid media, the bait LS2b protein was not toxic when expressed in yeast.

The interaction between CMV LS2b bait protein and library prey fusion proteins brought the DNA-binding domain (BD) and activating domain (AD) into proximity to activate the transcription of four independent reporter genes. Therefore, yeast grew on plates that lacked four amino acids with strong resistance of AbA and ability to turn the colonies blue. After 5 days of incubation, the mating production was plated on 50 plates, and high stringency selection patched out all the blue colonies growing on double-dropout agar plates with X-α-Gal, AbA and without tryptophan and leucine (DDO/X/A) onto quadruple-dropout agar plates with X-α-Gal, AbA and without adenine, histidine, tryptophan, and leucine (QDO/X/A). We collected 150 colonies, which were named 2b binding protein (2bBP, ranging from 1 to 150), for further sequencing. All plasmids were verified by DNA sequencing (Sangon Biotech). The sequencing results revealed that all plasmids contained inserts of parts or full-length ranging in size from 91 to 1881 bp. We compared and aligned all the sequences to those previously characterized in NCBI GenBank. The screening results of *Arabidopsis* cDNA libraries identified and classified 12 LS2b interacting proteins (S1 Table), including two uncharacterized proteins (2bBP19 and 2bBP78).

### Verification of CMV LS2b interaction with full-length binding partners using the yeast two-hybrid screen system

**Fig 1:**
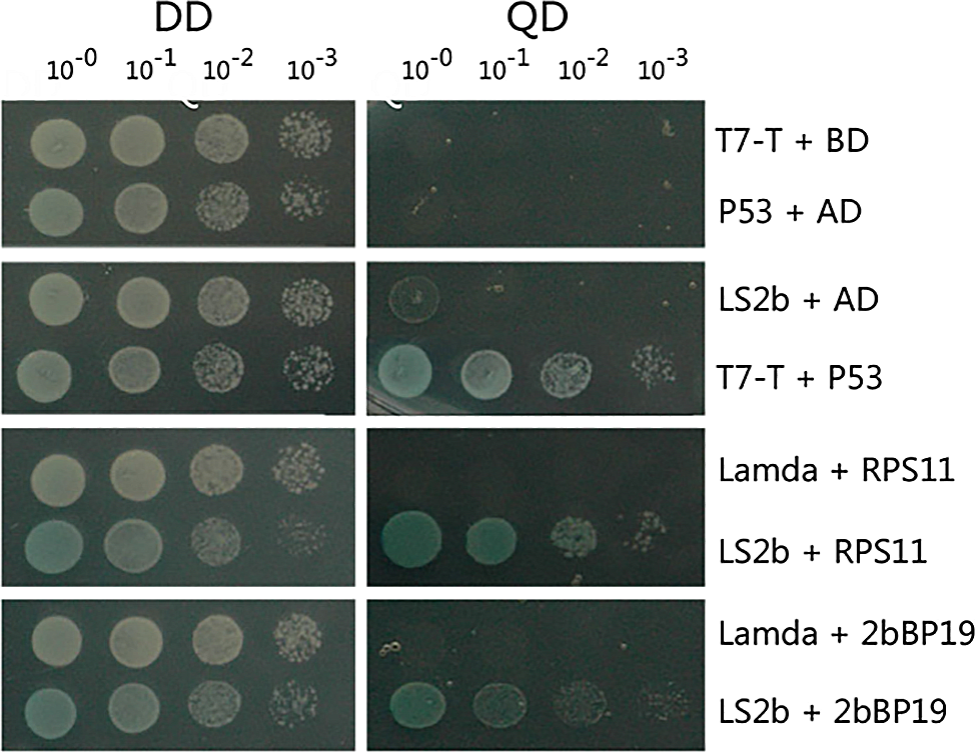
Interaction between bait LS2b protein and full-length prey protein was re-examined using yeast two-hybrid screening by DDO and QDO selection. Gradient dilutions of transformation mix were spread respectively on DDO/X and QDO/X/A plates which were cultivated for 5 days at 30°C. Yeast colony contained vectors of pGADT7-RPS11 with pGBKT7-LS2b and pGADT7-2bBP19 with pGBKT7- LS2b were showed on QDO/X/A plates. Vector pGADT7-T with pGBKT7-p53 and pGADT7 with pGBKT7-Lamda were used as positive and negative controls respectively.

By DNA sequencing before transformation into yeast, constructs containing full-length LS2b binding partners were authenticated. The bait protein was expressed as a fusion to the Gal4 DNA-binding domain (DNA-BD), whereas libraries of prey proteins were expressed as fusions to the Gal4 activation domain (AD). Therefore, when bait and prey fusion proteins interacted, the DNA-BD and -AD were brought into proximity to activate the transcription of independent reporter genes. With pGADT7 and pGBKT7, colonies grew on DDO plates. With the interaction between AtRPS11 and LS2b, At2bBP19 and LS2b colonies grew on DDO and QDO plates. Yeasts co-translated of pGADT7-T with pGBKT7-P53 as a positive control grew on DDO and QDO plates (Fig 1). Empty vector pGADT7 co-translated with pGBKT7-RPS11 and pGBKT7-2bBP19 only grew on DDO plates and not on QDO plates, similar to the empty vector pGBKT7 with pGADT7-LS2b. This finding suggested that RPS11 and 2bBP19 were indispensable binding partners with LS2b in the interaction system on QDO plates. After spreading the transformation dilutions of pGBKT7-RPS11 with pGADT7-LS2b on the QDO plates, visible yeast colonies began to appear at the fourth day; this rate was the fastest among all LS2b interacting partners (five days for all others; results not shown). This finding suggested that RPS11 recruitment of the LS2b binding partner was the most efficient at the subcellular level. Therefore, RPS11 was the strongest binding partner of LS2b, and we selected RPS11 for further investigation.

### LS2b interacts with NbRPS11 *in vitro* and *in vivo*

**Fig 2:**
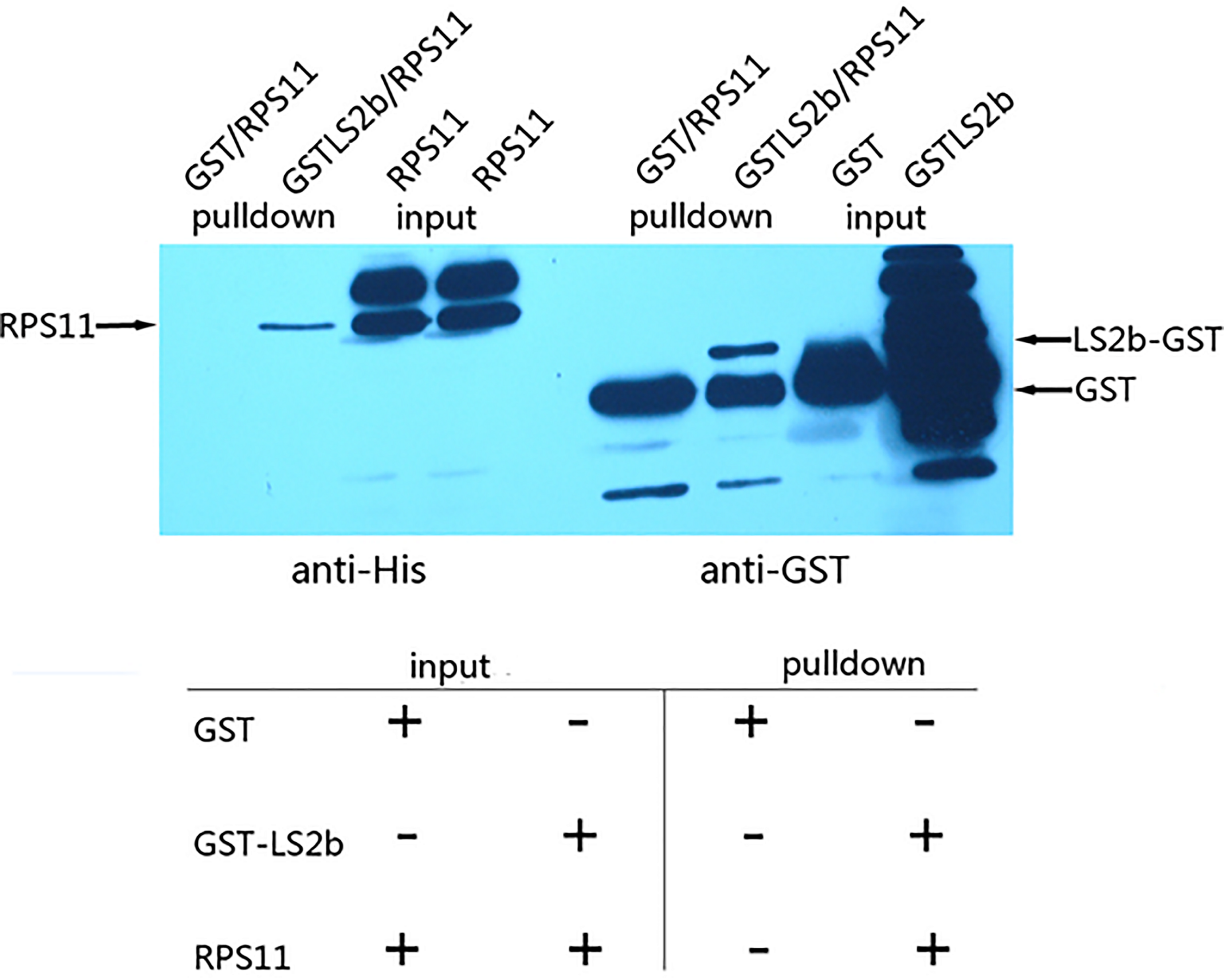
GST pull-down assay. GST and GST-LS2b fusion proteins incubated with cell lysates expressed His-RPS11 in tubes were the put-in samples transformed onto each right side of the membrane. Glutathione beads added to bind GST and GST-LS2b were washed down, proteins eluted from the beads were the pull-down samples transformed onto each left side of the membrane. The presence of RPS11 was detected by immunoblot with anti-HIS antibody. The presence and expression of GST and GST-LS2b was confirmed by immunoblotting with anti-GST antibody.

For pull-down assay, we placed GST and GST-LS2b bait protein into a column incubated with HIS-tagged prey protein RPS11. With the strong binding capacity of Glutathione HiCap Matrix to GST-fusion protein and the specific affinity between GST-LS2b and RPS11, GST-LS2b captured the binding partner RPS11 and formed a binding complex. We performed an immunoblot assay of the original samples and the pull-down samples by both anti-HIS and anti-GST antibodies onto one membrane to demonstrate the integrity in a clear display. As shown in Fig 2, we tested and verified the existence of HIS-tagged RPS11 in bead elution buffer of the lane-loaded GST-LS2b protein by anti-HIS immunoblot. As the control, no detectable trace of RPS11 was observed in GST-loaded bead elution buffer. We were aware of the overloaded lane. Initially, each lane was equally loaded for qualitative and quantitative measurements of the LS2b affinity to RPS11. LS2b directly interacted with RPS11 *in vitro* based on the equivalent RPS11 of both GST and GST-LS2b pre-incubated samples, in addition to the evidence that GST-LS2b but not GST captured the binding partner RPS11. A nonspecific band was only shown on the lane of GST and GST-LS2b pre-incubated input samples but not in the incubated elution pull-down samples. This finding illustrated that GST-LS2b specifically captured the binding partner RPS11 and was incapable of binding other nonspecific proteins. GST pull-down assay for interaction of RPS5 and LS2b as homologous control. The pull-down assay of LS2b, which interacted with another binding partner 30S ribosomal protein subunit S5 (RPS5), was performed using the same methods. The results shown in S2 Fig summarize the specific and strong interaction between RPS5 and CMVLS2b.

**Fig 3:**
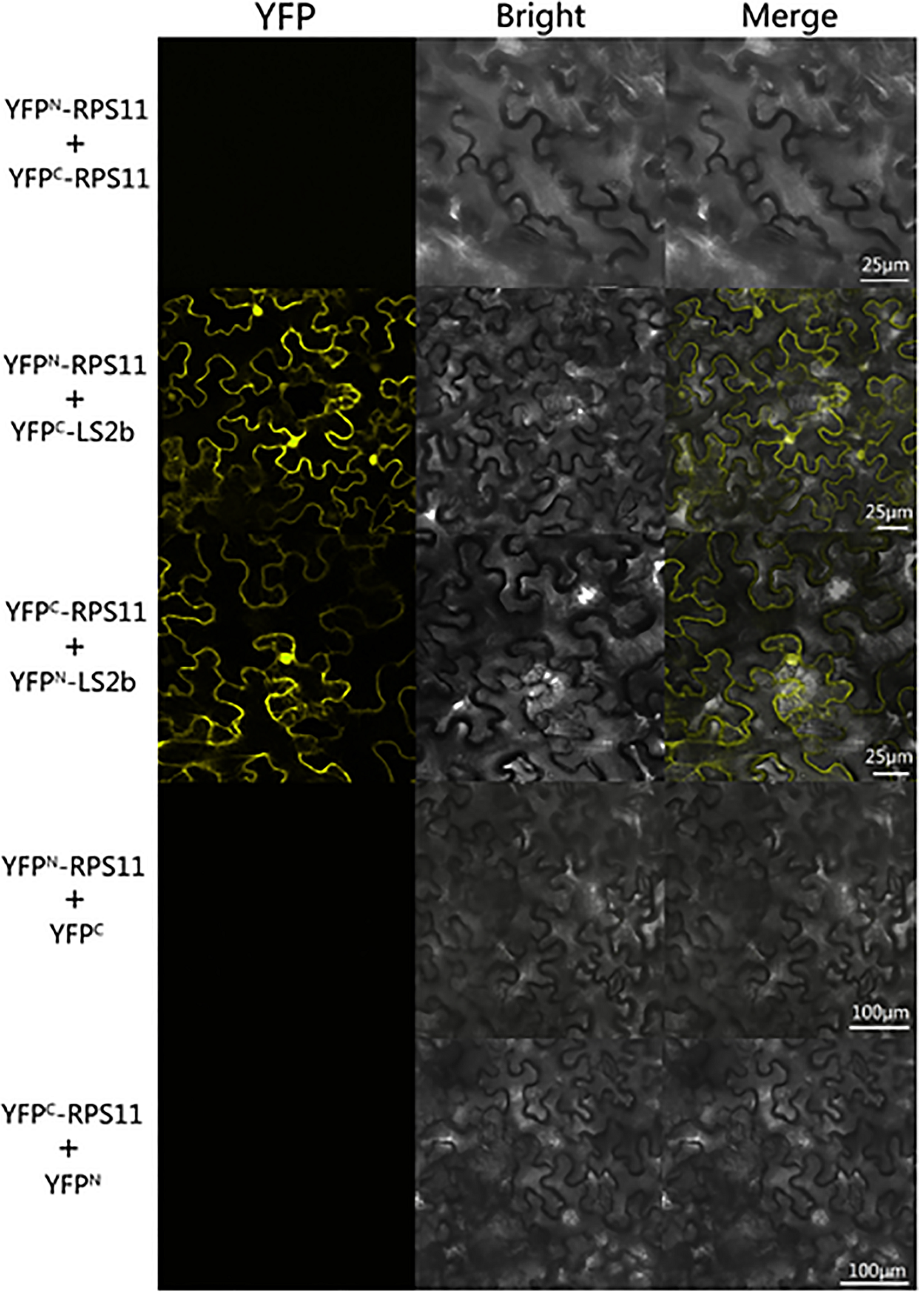
Bimolecular fluorescence complementation (BiFC) assay to test interaction of RPS11 with LS2b proteins *in vivo*. *N*. *benthamiana* was co-agroinfiltrated to transiently express combination of pSP-YCE-RPS11 and pSP-YNE-LS2b, pSP-YNE-RPS11 and pSP-YCE-LS2b. co-agroinfiltrated to transiently express pSP-YNE-RPS11 and pSP-YCE-RPS11 as self-interaction control. Also shown as controls in which the unfused YFP fragments (YFP^C^ and YFP^N^) were co-expressed with other of the RPS11-YFP fusion proteins. Cells in infiltrated tissues were imaged at 3 days post agroinfiltration by confocal scanning laser microscopy for YFP fluorescence and under bright field microscopy. The bar represented 25μm and 100μm.

To confirm the interaction of LS2b with NbRPS11 in living plant cells, we constructed plasmid-encoding fusion proteins of RPS11 and LS2b with either the N-terminal or C-terminal domain of a split yellow fluorescent protein, termed YFP^N^-RPS11, YFP^C^-RPS11, YFP^N^-LS2b, and YFP^C^-LS2b, respectively. Results of the BiFC assay showed that yellow fluorescence complementation via the reassembly formation of a bimolecular fluorescent complex partially composed of N-terminal and C-terminal fragments of YFP was caused by the interaction between RPS11 and LS2b proteins. No detectable fluorescence was observed in microscopic imaging of *N*. *benthamiana*. Cells within the leaf patches were co-infiltrated with YFP^N^-RPS11 and YFP^C^-RPS11, which revealed no self-interaction of the RPS11 protein *in vivo* (Fig 3). In cell cytoplasm that transiently co-expressed both combinations of YFP^N^-RPS11 and YFP^C^-LS2b and YFP^C^-RPS11 and YFP^N^-LS2b, strong YFP fluorescence signals were detected, whereas no fluorescence was detected in cytoplasm transiently co-expressing both combinations of YFP^N^-RPS11 and YFP^C^ and YFP^C^-RPS11 and YFP^N^, as the controls without binding partner LS2b protein. This observation confirmed the strong and specific interaction between NbRPS11 and LS2b *in vivo*.

### Knockdown of NbRPS11 decreased LS-CMV infectious viral RNA replication and viron accumulation

We used TRV-based gene silencing vector to knockdown NbRPS11 transcription. Semiquantitative RT-PCR was used to test for the NbRPS11 mRNA of plants inoculated with TRV2-RPS11 and control plants inoculated with TRV2. TRV2-RPS11 inoculation induced barely discernible symptoms in *N*. *benthamiana*, but NbRPS11 mRNA in systemic leaves of TRV2-RPS11 inoculation plants was remarkably lower than that in the TRV2 inoculation control group at 7-day post-inoculation (S3 Fig). We selected three independent plants with the same condition as a parallel control for each sample. No electrophoretic bands were observed in the 18th cycle NbRPS11 mRNA PCR amplification of either TRV2-RPS11 sample or TRV2 control. Electrophoretic bands of TRV2 control samples began to appear in the 21st cycle, but not in the TRV2-RPS11 samples. Distinct differences of electrophoretic bands between TRV2-RPS11 samples and TRV2 control were observed in the 24th cycle. RT-PCR for the NbRPS11 mRNA amplification of TRV2-RPS11 and TRV2 control samples in both the 21st and 24th cycles revealed that the transcriptional level of RPS11 was reduced by TRV-based gene silencing vector. We used the mRNA of endogenous β-actin amplified at the 24th cycle to compare as a loading control. The results of semiquantitative RT-PCR clearly revealed the RPS11 down-regulating effect of TRV2-RPS11 inoculation.

**Fig 4:**
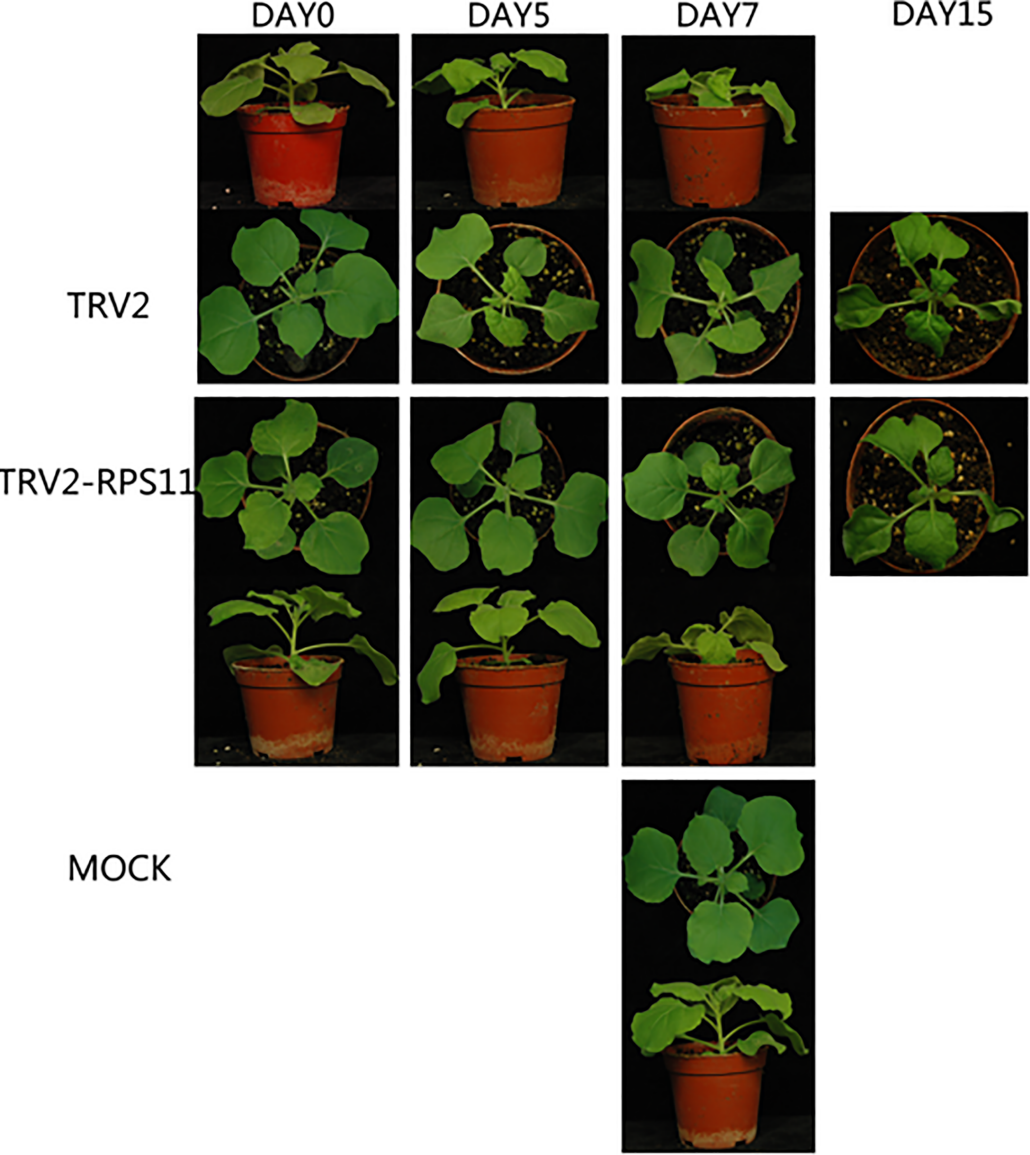
Knockdown of NbRPS11 decreased LS-CMV infectious viral RNA replication (A) Northern blot hybridization analysis of infectious viral RNA agroinfiltrated *N*. *benthamiana* 7 days after TRV2-RPS11 inoculation plants (lane 1–3) and TRV2 inoculation control plants (lane 4–6). Ethidium bromide stained rRNA from the same volume of each sample is shown below each lane. (B) Northern blot hybridization analysis of CMVΔ2b agroinfiltrated *N*. *benthamiana* 7 days after TRV2-RPS11 inoculation plants (lane 4–6) and TRV2 inoculation control plants (lane 1–3). (C) Western blot hybridization analysis of GFP accumulation 3 days post-inoculation of TRV2-RPS11 plants and conrol. Infectious viral RNA accumulation was analyzed by immunoblotting of testing accumulation of LS309-GFP of RPS11 downregulated plants (lane 1–3) and control plants (lane 4–6) using anti-GFP antibody. (D) GFP accumulation of PVX-GFP inoculation on RPS11 downregulated plants as comparative control showed at down panel. Ponceaus staining of rubisco protein was used to monitor equivalence of protein loading.

*Potato virus X* (PVX) is a typical member of the *Potexvirus* family. We used vector PVX-GFP as negative control of pCB301-LS109, pCB301-LS209, and pCB301-LS309∆CP-GFP. To harbor infectious viral mutants that could not express the 2b protein, we coagroinfiltrated pCB301-LS109, pCB301-LS209Δ2b (LS2b gene knockout mutant), and pCB301-LS309. We used this CMVΔ2b mutant as a negative control to verify the specificity of LS2b protein. Northern blot hybridization results of RNA1 (R1), RNA2 (R2), RNA3 (R3), and RNA4 (R4) showed that the replication of each single-stranded, positive-sense RNA declined in varying degrees by the down-regulated RPS11 (Fig 4A). Viral RNA replication level of CMVΔ2b mutant on TRV2-RPS11 and TRV2 plants was similar (Fig 4B). Northern blot hybridization results showed the separation of the bands of RNA1 and RNA2Δ2b because of the LS2b gene knockout. Therefore, viral RNA replications were influenced by RPS11 knockdown requiring essential existance of LS2b protein. Additionally, the knockdown of RPS11 effect on viral infection was not a consequence of a general reduction in protein synthesis but was because of the specificity of CMVLS2b expression. Thus, the difference of CMV infection between TRV2-RPS11 and TRV2 plants was due to the different levels of suppressor and host protein interaction affected by down-regulated RPS11 expression. The results of the western blot hybridization analysis of GFP expressed by CMV viral vector were consistent with the reduction in infectious viral RNA accumulation illustrated by GFP accumulation with the down-regulated RPS11 (Fig 4C). Accumulation of GFP expressed by PVX viral vector revealed that the accumulation of PVX RNA replication was not affected by the down-regulated RPS11 (Fig 4D).

**Fig 5:**
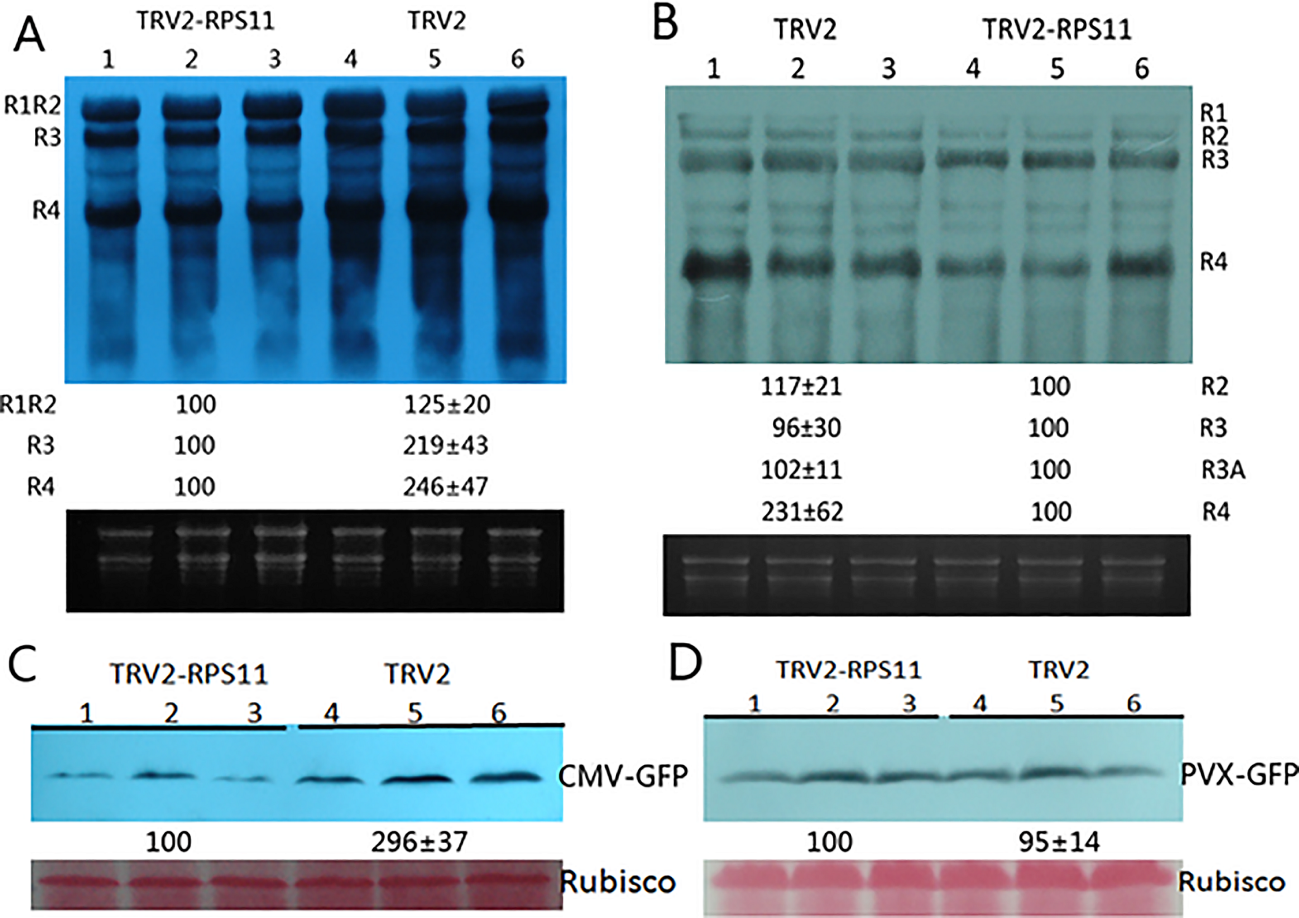
Viral symptoms of LS-CMV infected on systemic leaves at 7 dpi TRV2-RPS11 *N*. *benthamiana* plants and TRV2 controls. Symptom photos were taken at respectively 5 days, 7days, 15days after CMV mechanically infection. Mock was wild *N*. *benthamiana* plant 7 dpi TRV2-RPS11 without CMV infection.

**Fig 6:**
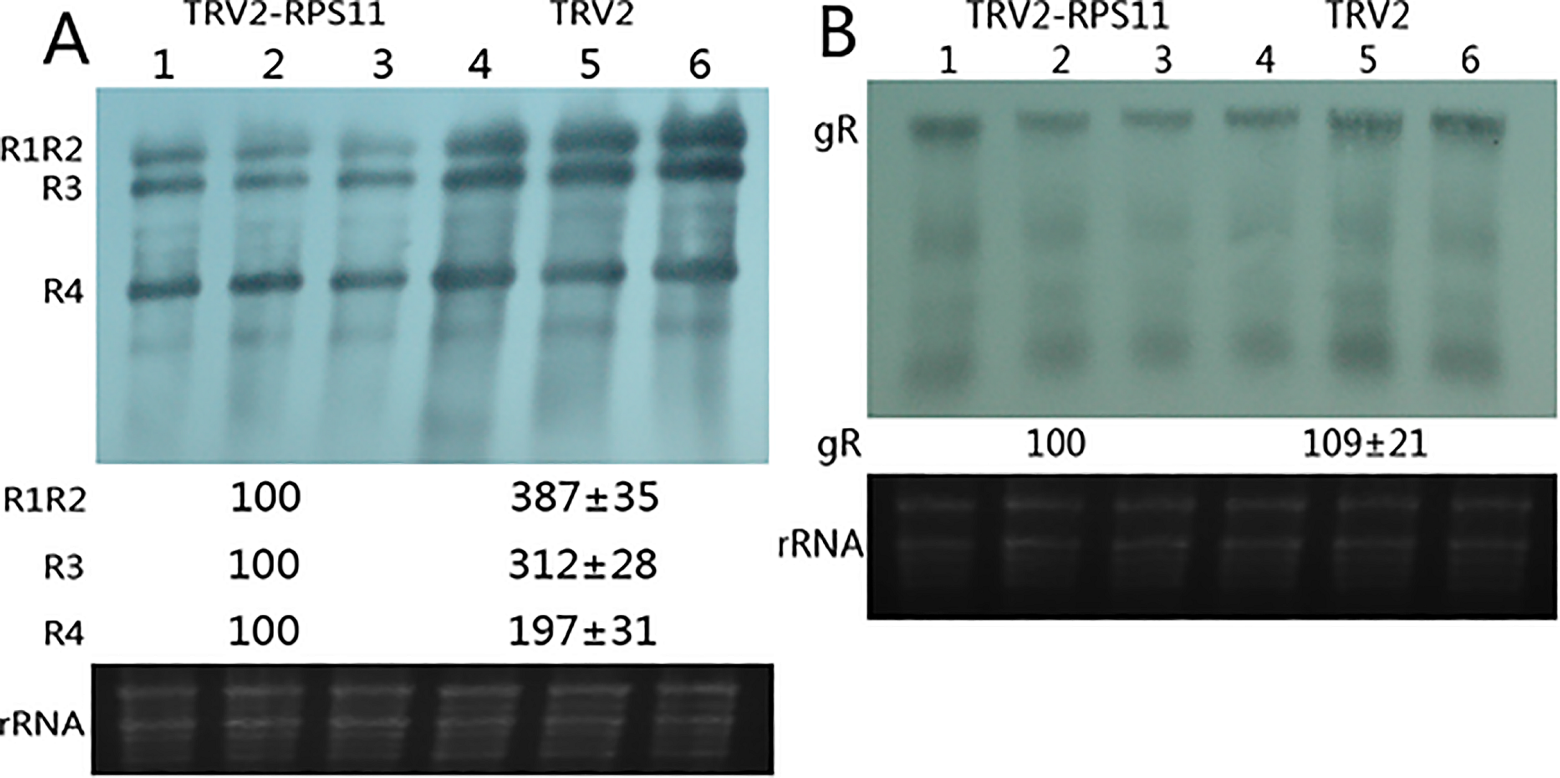
Northern blot hybridization analysis of 7 days post-infection of CMV(A) and ToMV(B) on RPS11 downregulated plants. CMV and ToMV were mechanically infected on *N*. *benthamiana* 7 days after TRV2-RPS11 inoculation plants (lane 1–3) and TRV2 inoculation control plants (lane 4–6). Equal loading of lanes of the northern blot was checked by ethidium bromide staining revealing rRNA bands showed as lower panel.

We performed Northern blot hybridization analysis of CMV and *Tomato mosaic virus* (ToMV) infection leaves at 7-day post-inoculation to investigate CMV infection and systemic accumulation of RPS11 downregulated plants. ToMV, a member of the tobamovirus genera, is genetically related to CMV. Curly leaf symptom and mosaic disease started to appear since the 4-day post-infection with TRV2 control plants, but was not observed with TRV2-RPS11 plants. Typical CMV disease symptoms were observed on the upper leaves at 7-day post-infection of both samples (Fig 5). The symptom appearance of TRV2-RPS11 developed two days later than that of TRV2 control. Thus, knockdown of RPS11 postponed symptom appearance of CMV infection. Accumulation of CMV RNAs was obviously reduced in TRV2-RPS11 inoculation plants compared with that of TRV2 control. CMV was significantly more sensitive to down-regulated RPS11 than ToMV. Knockdown of RPS11 did not inhibit the systemic movement of ToMV to non-inoculated cells in *N*. *benthamiana*. However, TRV2-RPS11 treatment inhibited CMV accumulation in non-inoculated cells (Fig 6). Northern blot hybridization analysis results revealed that down-regulated RPS11 affected CMV accumulation (Fig 6A). Upon knockdown of RPS11, LS-CMV had lower accumulation levels at 7 dpi in TRV2-RPS11 than in TRV2 control, whereas accumulation of ToMV remained the same as TRV2 control at 7 dpi (Fig 6B). Thus, exogenous infection was influenced by the endogenous protein RPS11.

### Knocking down the expression of NbRPS11 decreased gene silencing suppressor activity of CMV2b protein

**Fig 7:**
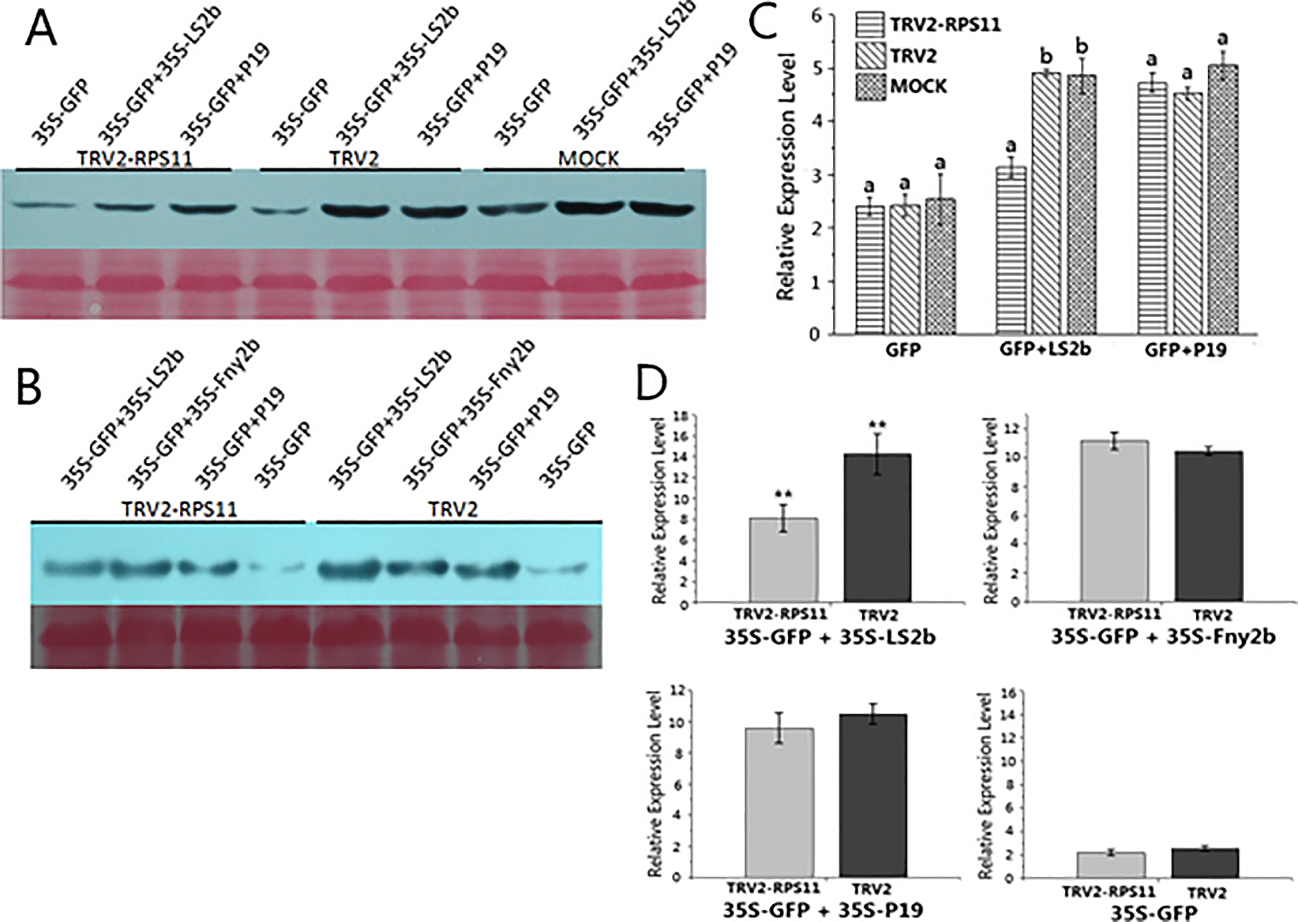
The gene silencing suppressor activity of CMV2b protein was reduced by the RPS11 knockdown. (A) Western blot hybridization analysis of gene silencing suppressor activity of CMVLS2b compared to P19 protein on RPS11 knockdown plants. Total protein samples were extracted 3 days post inoculation of gene silencing suppressor. (B) Further GFP silencing suppressor activity test of LS2b protein was performed with comparison of both P19 and homologous Fny2b proteins. Ponceaus staining of rubisco protein was used to monitor equivalence of protein loading. (C) Quantification for relative GFP accumulation of gene silencing suppressor affected on RPS11 knockdown plants and controls. (D) Comparison of each gene silencing suppressing activity on RPS11 knockdown plants and control. Each value is the mean of three replicates and vertical bars are SD. For a given parameter, means with different letters in C are statistically different at P<0.05. *Asterisks* in D indicate a significant difference between treatments and the control at P<0.01 (**) according to Student–Newman–Keuls test.

Given that the CMV 2b protein primarily functions as a gene silencing suppressor, we analyzed this factor in the case of RPS11 knockdown *N*. *benthamiana*. We used typical tombusvirus P19, which is a widely used efficient gene silencing suppressor, as the control. Preliminary GFP-silencing suppressor activity tests of CMVLS2b protein on RPS11 knockdown plants, TRV2 control, and mock plants were performed with comparison of transiently co-expressing P19 and non-VSR control plants. Mock samples comprised wild *N*. *benthamiana* plants inoculated with sterile distilled water alone. GFP accumulation in TRV2-inoculated plants displayed no discernible differences with the mock-inoculated plants (Fig 7A). Additionally, GFP expression was silenced by the host antiviral defense. Without transiently co-expressing LS2b or P19 protein that inhibited RNA-induced silencing complex activity, GFP silencing was considered efficient. GFP accumulation in TRV2-RPS11 co-inoculated with LS2b was distinctly lower than that of TRV2-inoculated, which suggested stronger host antiviral silencing activity in TRV2-RPS11 inoculation plants. Hence, silencing suppressed activity of LS2b was significantly reduced under the influence of down-regulated RPS11. Quantification for relative GFP accumulation of LS2b was significantly affected by RPS11 knockdown compared with control plants (Fig 7C). Gene silencing suppressing activity of LS2b and P19 protein on TRV2 control and mock plants was coincident.

A further GFP-silencing suppressor activity test of CMV2b protein was performed with a comparison of both P19 and homologous CMVFny2b proteins. The subcellular localization abilities of Fny2b and LS2b proteins are differently distributed due to differential NLS sequences. Compared with Fny2b protein, 2b genes from milder subgroup II strains Q or LS cause symptom-like developmental defects [4] [9]. In this work, GFP-silencing was efficiently suppressed in all VSR-expressing cells compared with non-VSR control (Fig 7B). Western analysis of GFP accumulation transiently co-expressing with P19 or CMVFny2b displayed no discernible differences between TRV2-RPS11 inoculated plants and TRV2 controls. Only GFP accumulation of co-expressing with LS2b was affected by down-regulated RPS11, which showed subgroup specificity (Fig 7D).

## Discussion

CMV 2b, a determinant for virulence, interacts with host factors during pathogenesis to facilitate viral infection, is involved in local and systemic movements of CMV, and inhibits host defense through RNA silencing suppression. In this work, 12 host proteins were identified as CMV LS2b binding partners by the yeast two-hybrid screen system from the *Arabidopsis* *thaliana* cDNA library. LS2b-interacting partner proteins were distributed throughout the cells from the center nucleus, endomembrane system to the cytosol and organelles such as ribosomes, chloroplast thylakoid membrane, and mitochondrial envelope. This finding showed that many host proteins regulate the multi-functional CMV 2b protein. LS2b-interacting partner proteins have various functions: 30S ribosomal protein is the key to protein synthesis, HSP40 and DJA6 belong to the family of heat shock proteins, several proteins are involved in RNA synthesis regulation and cytosolic membrane trafficking, and some were uncharacterized proteins with unknown functions. Among these proteins, RPS11 was selected for further studies. The binding partner AtRPS11 interacting with LS2b was confirmed by the full-length yeast two-hybrid system. Verification that CMV LS2b interacted with full-length binding partners suggested that full-length RPS11 effectively recruited LS2b as a binding partner. Full-length RPS11 showed high abundance and rapid growth among all LS2b interacting partner clones; therefore, RPS11 is the strongest binding partner of LS2b.

Direct interaction of NbRPS11 with LS2b *in vitro* was confirmed by the GST pull-down assay. Additionally, we performed the BIFC assay to verify the RPS11–LS2b interaction *in vivo*. We assumed that the direct interaction of NbRPS11 with CMVLS2b might function as a bridge to connect plant–virus interactions. Localization of CMV 2b to the nucleus depends on its interaction with host *A*. *thaliana* karyopherin-like protein [11]. Immunoprecipitation assays of CMV 2b with AGO1 revealed that the two proteins interacted *in vivo* and *in vitro*. The 2b protein blocks AGO1 cleavage activity to inhibit RNA silencing [4]. The formation of *A*. *thaliana* necrotic lesions is associated with the increase in hydrogen peroxide as a result of the interaction between catalase (CAT3) and CMV 2b protein [12]. Research continues to show that interactions between host proteins and 2b protein affect the function of viral infection.

Despite much research aimed at determining the biochemical changes at the protein or RNA level in infected plants, the mechanisms of infection remain poorly understood. TRV-based gene silencing vectors were used to produce a transgenic genotype susceptible to CMV [13]. We used TRV-based gene silencing vector to knockdown NbRPS11 transcription. Down-regulation of RPS11 mRNA transcription was confirmed by semiquantitative RT-PCR, which was extremely important as the foundation for the following tests. Viral RNA accumulation levels in RPS11 knockdown plants were detected by northern blot hybridization, and the results showed that infectious viral RNA replication decreased and CMV infection declined in RPS11 down-regulated plants. The results of CMVΔ2b RNA replications illustrated that the influence of virus on RPS11 knockdown plants required essential existence of LS2b protein. Accumulation of viral coat protein (CP) illustrates the infectious viral RNA replication in host cells. To investigate CMV infectious viral RNA replication in RPS11 knockdown plants, we co-inoculated pCB301-LS109, pCB301-LS209, and pCB301-LS309∆CP-GFP to harbor infectious clones in the inoculation plants. The CP gene sequence was replaced by an EGFP gene sequence in pCB301-LS309∆CP-GFP plasmid, and the accumulation of GFP protein expression illustrated the infectious viral RNA replication of CMV. The virus lost cell-to-cell movement, as a result of the inability to completely assemble a virion without a CP. Therefore, viral RNA of CMV only existed and replicated in local cells in the range of agroinfiltration patches. Western blot hybridization results of CMV-GFP and PVX-GFP verified that infectious viral RNA replication decreased in RPS11 knockdown plants. The results indicated that the down-regulated RPS11 specifically affected CMV infectious viral RNA replication but not that of PVX. Clear evidence showed that the knockdown of RPS11 inhibited CMV replication and accumulation by recruiting viral LS2b to interact. Knockdown of RPS11 disrupted the balance of dynamic equilibrium of interaction with viral LS2b, which facilitated viral assembly in the host cells.

Northern blot hybridization analysis of CMV and ToMV infection showed that CMV infection and systemic accumulation were affected in RPS11 down-regulated plants. The resistant genes of infected plants to CMV and ToMV have been identified, and they are syntenic genes with similar resistance [14]. The ToMV infection control was intended to exclude the effect of viral infection on physiological changes. The symptoms in TRV2-RPS11 appeared milder and developed later than that those in TRV2 control. Therefore, knockdown RPS11 had a postponed symptom appearance and desensitized CMV infection. Knockdown of RPS11 inhibited CMV replication, and accumulation which revealed that RPS11 levels were positively correlated with viral LS2b function. RNA interference constructs were used to silence the sequence region of the AC1 viral gene of *Bean golden mosaic virus* (BGMV) and highly resistant transgenic plants were generated [15]. Some single resistance genes have been characterized. For example, when ssi2 gene down-regulated, JA and SA pathways appear more resistant to the virus [16]. In this paper, the endogenous protein RPS11 influenced the exogenous infection. Therefore, the strategy of plant virus resistance begins, in part, with the regulation of the transcription of the endogenous binding partner protein.

Gene silencing suppressor activity of CMV 2b protein was also affected by RPS11 down-regulation. The 2b protein suppresses RNA silencing by specifically inhibiting AGO1 cleavage activity in RISC reconstitution [4]. The results in Fig 7 show that 2b-silencing suppression activity was compromised by RPS11 knockdown and suggest that 2b protein recruited RPS11 by direct interaction to facilitate gene silencing suppressor activity. The silencing suppressor activity test with comparison of homologous CMVFny2b proteins suggested that inhibition of silencing suppression activity was directly related to LS2b. Fny2b functions the same way as LS2b; gene silencing suppressor activity of Fny2b protein was not affected by RPS11 down-regulation, and therefore, RPS11 regulated gene silencing suppressor activity by direct interaction with LS2b, not through affected steps. The results further substantiated the gene silencing suppressor activity of CMV2b protein as influenced by interaction between viral LS2b and endogenous RPS11. Additional siRNA immunoblot analysis is required to determine which step of the endogenous silencing pathway is associated with RPS11. Collectively, the translation level of endogenous RPS11 positively correlated with the viral LS2b protein functions through the interaction between the two proteins.

RPS11 down-regulated transgenic plants may obtain potential resistance against CMV. Therefore, we intended to establish RPS11 knockdown transgenetic *Arabidopsis* mutant plants using the *Agrobacterium* floral dip method. However, RPS11 gene normal expression is indispensabe to *Arabidopsis* plants; as a result, germinated seedlings of transgenetic mutant plants did not grow on the kanamycin selection medium. Notably, RPS5 knockdown transgenetic *Arabidopsis* grew on the kanamycin selection medium and transferred onto the solid medium to grow, bloom, and successfully subculture. Further tests of CMV infection and replication will be performed on RPS5 knockdown transgenetic *Arabidopsis* mutant plants.

CMV2b functions as a symptom determinant according to its gene silencing suppressor activity and effect on viral accumulation. Endogenous NbRPS11 can be defined as a determinant for host-viral regulating functions because of its interaction with CMV2b. We found that host RPS11 affects gene silencing suppression by interacting with viral 2b protein, which is important information to elucidate the mechanisms for host–virus pathogenicity.

## Methods and materials

### Plasmid constructions

For BIFC assays, the full length NbRPS11 fragment was amplified by the primers 5’ F1-*BamH*I (GGGATCCATGAATGGTGTCTCTCGT CA) and 3’ R-*Sac*I (TTGTCGACCACCCGACGTTTCCT) with the sequenced plasmid pUC18-NbRPS11 as the template. Virus-induced gene silencing vectors pTRV-RNA1 and pTRV-RNA2 were obtained from a former construction. Total length NbRPS11 fragment was amplified by the primers 5’ F1-*BamH*I (ATGGATCCATGAATGTTTATTCACCATATAATG) and 3’ R-*Sac*I (ATGAGC TCCCCGGGTTAGATGCGCGCTTATTTG) with the sequenced plasmid pUC18-NbRPS11 as the template. The full-length NbRPS11 fragment was digested with restriction endonucleases *BamH*I and *Sac*I and then cloned into pTRV-RNA2 plasmid that was previously digested with the same restriction endonucleases. We transformed VIGS vectors pTRV-RNA1, pTRV-RNA2, and pTRV-RNA2-NbRPS11 into GV3101 agrobacteria competent cells for coagroinfiltration. Plasmids pCB301-LS109, pCB301-LS209, and pCB301-LS309∆CP-GFP were previously constructed with T-DNA vectors containing LS-CMV RNA1, RNA2, and RNA3 to harbor infectious clones in the inoculated plants.

### Yeast two-hybrid system was used to identify proteins that interact with 2b protein from the *Arabidopsis* cDNA library

The Matchmaker Gold Yeast Two-Hybrid System (Clontech, USA) was used to screen the interaction of the host proteins with the bait protein LS2b. The *Arabidopsis* cDNA library (BioTech, GER) was prepared in plasmid pGADT7-Rec and transformed into yeast strain Y187. The bait plasmid pGBKT7-LS2b [17] was transformed into yeast strain Y2H.Gold. To confirm that the bait protein LS2b did not autonomously activate the reporter genes without a prey protein, we transformed the pGBKT7-LS2b construct into previously prepared competent Y2HGold cells. Gradient dilution of the transformation mixture was spread onto SD plates lacking tryptophan (SD-TRP) and SD plates containing 5-bromo-4-chloro-3-indoyl-α-d-galactopyranoside (X-α-Gal) and Aureobasidin A (SD-TRP/X/A). The mixture was cultivated at 30°C for 3 days as a bait autoactivation test. Control mating was performed before screening the library. We used pGBKT7-53 as the positive control bait plasmid in Y2HGold yeast bait strain and pGADT7-T as the positive control prey plasmid in Y187 yeast prey strain. pGBKT7-Lam was used as the negative control bait plasmid in Y2HGold yeast bait strain and pGADT7 as the negative control prey plasmid in Y187 yeast prey strain. We used the Yeastmaker Yeast Transformation System according to the small-scale protocol to perform the transformations that were spread onto the SD-TRP and SD plates lacking leucine (SD-Leu) growing at 30°C for 3 days. The colonies of each type were selected for the following small-scale mating procedure and were incubated with shaking at 200 rpm at 30°C overnight. Gradient dilutions were spread onto SD-Leu, SD-Trp, SD/–Leu/–Trp (DDO), and DDO/X/A plates, which were subsequently incubated at 30°C for 5 days. After testing for autoactivation and control experiments, the two-hybrid library screening using the yeast mating was performed according to the protocol. We combined the library prey strain with the prepared concentrated overnight culture of the bait strain for mating of 24 h, with 30 rpm slowly shaking at 30°C in a sterile 2 L flask incorporated with 2×YPDA liquid medium with kanamycin. From the mated culture, we spread gradient dilutions on DDO/X/A 50 plates of 150 mm size placed in an incubator at 30°C for 5 days, and patched out all the blue colonies growing on DDO/X/A onto QDO/X/A with higher stringency. All QDO/X/A positive colonies plasmids were extracted using a TIANprep Yeast Plasmid DNA Kit for further analysis.

### Verification of positive interactions and full-length prey protein interaction

We performed several steps before sequencing the positive clones to isolate the library plasmid that activated the reporters and distinguished genuine positive from false positive interactions. We used the Matchmaker Insert Check PCR Mix (Clontech, USA) to amplify cDNA prey library inserts from the pGADT7 plasmids extracted from the QDO/X/A positive colonies. We analyzed PCR products by electrophoresis on TAE agarose, selected the plasmids with multiple bands, and purified the QDO/X/A positive clones by three times repeated plate streaking blue colonies. Using the small-scale transformation, each of the selected prey in pGADT7 and LS2b bait protein in pGBKT7 was co-transformed into Y2HGold competent cells with empty pGBKT7 as the control. We spread gradient dilutions of the transformation mix on DDO/X and QDO/X/A plates. When an interaction was verified as genuine, the prey cDNA inserts were identified through sequencing using the Matchmaker AD LD-Insert screening amplifier and T7 sequencing primer. We transformed the plasmids with low-sequencing signal intensity prey cDNA inserts into DH5α*E*.*coli* competent cells and isolated the purified, highly concentrated plasmids for further sequencing.

For verification of the full-length of prey protein interacting with bait LS2b, the total length AtRPS11 fragment was amplified by the primers 5’ RPS11-*Sfil*-*BamH*I-F (ATGGCCATGGAGGCCGGATCCATGAATGGTGTCTCTCGTCA) and 3’ RPS11-*Sac*I-R (ATGAGCTCTCACACCCGACGTTTCCT) with the sequenced mRNA extracted from *Arabidopsis* RNA as the template. Total length At2bBP19 fragment was amplified by the primers 5’ 2bBP19-Stul-*EcoR*I-F (GAGGCCTGAATTCATGTCGTGT TTAGCCCTAGC) and 3’ 2bBP19-*SacI*-R (TTGAGCTCTCAATTGCACATATAGACTCC). The full-length AtRPS11 and At2bBP19 fragments were digested with restriction endonucleases, cloned into pGADT7 plasmids, and co-transformed into competent yeast cells with pGBKT7 containing LS2b. Gradient dilutions of the transformation mix were spread on DDO/X and QDO/X/A plates cultivated at 30°C for 5 days.

### *In vitro* pull-down assay analyses

The GST pull-down assay was performed using purified GST and GST-LS2b from the cell lysates to confirm the interaction of CMVLS2b with NbRPS11. HIS-RPS11 protein was expressed by pET28a vector in *E*. *coli*. BL21 (DE3) was purified using a Ni-NTA Purification Kit (MCLAB, USA). GST-tagged LS2b protein was expressed by pGEX4T-GST vector in *E*. *coli*. BL21 (DE3) was purified using a Glutathione Affinity Purification Kit (QIAGEN, GER). Before loading the lysate into the column with Glutathione HiCap Matrix slurry, resin was equilibrated with PBS-EW buffer. The lysate containing bait GST-tagged LS2b and GST control proteins was twice washed with PBS-EW buffer. TNGT buffer was added to dissolve the target proteins, and all flow-through elution fractions were collected for SDS-PAGE analysis.

For the pull-down assay, GST-tagged LS2b bait protein and GST control protein were mixed with His-tagged NbRPS11 prey protein sample and were equilibrated with TN1 buffer and Glutathione HiCap Matrix washed by TN1 buffer in tubes. The tubes were incubated by constant gentle inverting for 4 h at 4°C. The beads were then washed three times with TN1 buffer, gently mixed with TN2 buffer, incubated at 4°C for 10 min, and centrifuged. We collected the supernatant containing the protein complexes for western blot hybridization analysis.

The samples were incorporated with equal volumes of 2×SDS loading buffer and were denatured at 95°C for 10 min. Each sample was equally loaded per lane and separated by SDS-containing 15% polyacrylamide gel electrophoresis. After ponceau dyeing confirmation, the separated proteins were electrophoretically transferred to blot onto nitrocellulose transfer membranes (Whatman, UK) at 100 mA per membrane for 1 h. Membranes were blocked for 2 h in 1×TBS blocking buffer (20 mM Tris-HCl, 150 mM NaCl) containing 5% skimmed milk before incubating for 2 h in anti-His primary antibody to detect NbRPS11 and in anti-GST primary antibody to detect the GST and GST-LS2b proteins diluted (1:5000) in the blocking buffer. The membrane was washed by TBST buffer (0.1% Tween-20 added to 1×TBS buffer) three times in 10 min and by TBS once in 10 min and was subsequently incubated for 2 h with goat anti-rabbit IgG horseradish peroxidase-conjugated as the secondary antibody (1:5000 dilution). The antigen-antibody reaction was detected using a chemiluminescent Biotin-labeled Nucleic Acid Detection Kit (Beyotime) and visualized through developing liquid/fixing solution washed films.

### BiFC assays

The full-length NbRPS11 fragment was digested with restriction endonucleases *BamH*I and *Sac*I and then cloned into pSP-YNE and pSP-YCE plasmids that were previously digested with similar restriction endonucleases. The plasmids with the corresponding fusion proteins with YFP N-terminal and YFP C-terminal to the LS2b protein were previously cloned. All plasmids used for BiFC assays were separately transformed into GV3101 agrobacteria competent cells for coagroinfiltration. *Agrobacterium* cells containing construct combinations of pSP-YNE-RPS11 and pSP-YCE-RPS11; pSP-YCE-RPS11 and pSP-YNE-LS2b; pSP-YNE-RPS11 and pSP-YCE-LS2b; pSP-YCE-RPS11 and pSP-YNE; and pSP-YNE-RPS11 and pSP-YCE were selected by kanamycin, gentamicin, and rifampicin. These cells were centrifuged, washed, and dissolved in solution buffer (10 mM MgCl2, 10 mM MES, and 100 μM AC). *Agrobacterium* solution was measured and diluted to a final optical density at 0.6 of OD_600_ and coinfiltrated simultaneously into the fifth and sixth leaves of *N*. *benthamiana* plants. At 3-day post-inoculation, YFP fluorescence (excitation and emission maxima at 514 and 527 nm, respectively) was observed using a Leica SP5 confocal laser-scanning microscope (Leica Microsystems, German).

### Infectious viral RNA inoculations

The mRNA levels of RPS11 in the upper systemic leaves were examined using semiquantitative RT-PCR using primers F1 (ATGGATCCATGGGAAACATTCTGACCAATGGA) and R1 (ATGAGCTCCCCGGGTTAGATGCGCGCTTATTTG) at 7-day post-infiltration with pTRV-RNA2-NbRPS11 and pTRV-RNA1. pTRV-RNA1+ pTRV-RNA2 was set as the control. Total RNA of upper systemic leaves was extracted, and mRNA was amplified using random primer mixed with oligo (dT) primers. We sampled three independent plants with same condition as the parallel control and performed PCR amplification on 18, 21, and 24 cycles.

We injected patches of infectious viral RNA plasmids into systemically infected leaves at 7-day post pTRV-RNA2-NbRPS11 inoculation. Patches were obtained from the samples for northern blot and western blot hybridization analyses. The leaf tissue was pulverized in liquid nitrogen, and total protein was extracted from *N*. *benthamiana* agroinfiltrated patches by phosphate-buffered saline added 2% (v/v) β-mercaptoethanol. Each sample was equally loaded and separated by SDS-containing 15% polyacrylamide gels electrophoresis. After ponceau dyeing confirmation, separated proteins were electrophoretically transferred to nitrocellulose membranes at 100 mA per membrane for 1 h. The membrane was probed using the anti-GFP polyclonal serum to detect the GFP protein. The primary antibody binding was detected by goat anti-rabbit IgG horseradish peroxidase conjugate as the secondary antibody. Total RNA was extracted from *N*. *benthamiana* agroinfiltrated patches using TRIzol (Invitrogen-Life Technologies) according to the manufacturer’s instructions. The membrane was pre-hybridized with DIG Easy Hyb Granules at 45°C for 4 h and hybridized using probe with dilution buffer overnight at 45°C. The membrane was washed twice with 0.5×SSC buffer, each time for 15 min, and twice by 0.2×SSC buffer, each time for 15 min. After 1 h blocking at 25°C, anti-DIG AP conjugate antibody was incorporated into the blocking buffer and was incubated for another 1 h at 25°C. The DIG-labeled probe was detected using a DIG Luminescent Detection Kit (Roche). Quantitative analysis of infectious viral RNA replication was calculated by ImageJ software with the ‘‘intensity correlation analysis”.

### Virus infection

Activated agrobacteria containing plasmids pCB301-LS109, pCB301-LS209, and pCB301-LS309∆CP-GFP [17] were systemically infected leaves 7-day post pTRV-RNA2-NbRPS11inoculation. LS-CMV was transported from the infected *N*. *glutinosa* to *N*. *tabacum* plants for virus propagation and virus purification. Purified virions were mechanically inoculated onto systemically infected leaves at 7-day post pTRV-RNA2-NbRPS11inoculation at a concentration of 100 ng/μl using Carborundum as an abrasive. Total RNAs were extracted from the systemic leaf above the infected leaves at 7-day post infection. The protocols for RNA isolation, denaturation, electrophoresis, and transfer to membranes were as previously described. We used ImageJ software to quantize northern blot analysis results.

### Agroinfiltration patch assays for RNA silencing suppression

The vector p35S:GFP promoted by the constitutive Cauliflower Mosaic Virus 35S promoter was derived from the plasmid pBI121. Agrobacteriun carrying p35S:GFP singly and co-infiltrated respectively with p35S:LS2b, p35S:P19, and p35S:Fny2b [18] on systemically infected leaves at 7-day post pTRV-RNA2-NbRPS11inoculation. Patches were observed and recorded under UV light in darkroom using a Nikon BM-5 digital camera. Each patch was obtained from a sample at 3-day post inoculation for western blot hybridization analysis. The protocols for total protein isolation, electrophoresis, and immunoblot were as previously described.

### Statistical analysis

We used ImageJ to measure relative expression of GFP by quantizing western blot results, and RNA expression by quantizing northern blot results into numerical data. All the agroinfiltration experiments for gene silencing activity tests were repeated 3 times. The data were subjected to statistical analysis using SPSS 16.0. All the histograms were made by OriginPro 8.0. Each value is the mean of three replicates and vertical bars are standard deviation (SD). The statistical significance of the results was analyzed by the Student–Newman–Keuls test (SNK) at the 1 or 5% significance level.

## Supporting information

S1 Table Identification of CMV LS2b-interacting partners by yeast two hybrid screen system. Pate6, functions in transporter activity, locate in plasma membrane; LP1, functions in zinc ion binding, locate in cytosol, nucleus, phragmoplast; DUF220, domain of unknown function; DJA, HSP40, functions in protein folding, unfolded protein binding, heat shock protein binding, ATP binding, locate in chloroplast thylakoid membrane, chloroplast; KRS, functions in ATP binding, lysine-tRNA ligase activity, lysyl-tRNA aminoacylation, translation, tRNA aminoacylation for protein translation, locate in cytoplasm; AP22, functions in splicing factor, suppressor; A1E, functions in aldose 1-epimerase activity, galactose metabolic process, hexose metabolic process, carbohydrate metabolic process, locate in endomembrane system; RPS5, RPS11, functions in structural constituent of ribosome, translation, locate in cytosolic small ribosomal subunit, ribosome; 2bBP19, 2bBP78, *Arabidopsis thaliana* uncharacterized protein.
(DOCX)

S1 Fig Testing LS2b bait for autoactivation and toxicity on QD and DD medium plates. Confirmation of co-translation pGADT7-T with pGBKT7-P5 as positive control on QD medium plate. 1:10, 1:100,1:1000 gradient dilutions were spread on each one third of plates for illustration of co-translation ability.
(TIF)

S2 Fig GST pull-down assay for interaction of RPS5 and LS2b as homologous control. The presence of RPS5 was detected by immunoblot with anti-HIS antibody on left side of the membrane. The presence and expression of GST and GST-LS2b was confirmed by immunoblotting with anti-GST antibody on right side of the membrane.
(TIF)

S3 Fig Results of semiquantitative RT-PCR for the NbRPS11 mRNA of TRV2-RPS11 inoculation plants (lane 1–3) and TRV2 inoculation control plants (lane 4–6). The upper, middle, lower panels of NbRPS11 mRNA were respectively PCR amplified products of 18th cycle, 21st cycle and 24th cycle. The mRNA of endogenous β-actin amplified at 24th cycle served as a loading control.
(TIF)

## Abbreviations

CMV: *Cucumber mosaic virus*
RPS11: 30S ribosomal subunit protein S11
RPS5: 30S ribosomal protein subunit S5
CMVLS2b: 2b protein encoded by RNA2 from LS strain of CMV
CMVFny2b: 2b protein encoded by RNA2 from Fny strain of CMV
MP: movement protein
CP: capsid protein
ORF: open reading frames
BiFC: bimolecular fluorescence complementation
VIGS: virus induced gene silencing
rpm: round per minutes
GFP: Green Fluorescent Protein
YFP: Yellow Fluorescent Protein
dpi: days post inoculation
PVX: *Potato Virus X*
ToMV: *Tomato Mosaic Virus*
TRV: *Tobacco Rattle Virus*
BD: Binding Domain
AD: Activating Domain
X-α-Gal: 5-bromo-4-chloro-3-indoyl-α-d-galactopyranoside
AbA: Aureobasidin A
GST: Glutathione S-transferase
DDO/X/A: double-dropout agar plates with X-α-Gal, AbA without tryptophan, and leucine
QDO/X/A: quadruple-dropout agar plates with X-α-Gal, AbA without adenine, histidine, tryptophan, and leucine
SNK: Student–Newman–Keuls test
SD: standard deviation
