# Supplementary material for: The interaction between endogenous 30S ribosomal subunit protein S11 and Cucumber mosaic virus LS2b protein affects viral replication, infection and gene silencing suppressor activity
Source: PLoS One. 2017 Aug 14;12(8):e0182459. doi: 10.1371/journal.pone.0182459 (PMC5555695; doi:10.1371/journal.pone.0182459)
Supplement: S1 Table — Pate6, functions in transporter activity, locate in plasma membrane; LP1, functions in zinc ion binding, locate in cytosol, nucleus, phragmoplast; DUF220, domain of unknown function; DJA, HSP40, functions in protein folding, unfolded protein binding, heat shock protein binding, ATP binding, locate in chloroplast thylakoid membrane, chloroplast; KRS, functions in ATP binding, lysine-tRNA ligase activity, lysyl-tRNA aminoacylation, translation, tRNA aminoacylation for protein translation, locate in cytoplasm; AP22, functions in splicing factor, suppressor; A1E, functions in aldose 1-epimerase activity, galactose metabolic process, hexose metabolic process, carbohydrate metabolic process, locate in endomembrane system; RPS5, RPS11, functions in structural constituent of ribosome, translation, locate in cytosolic small ribosomal subunit, ribosome; 2bBP19, 2bBP78, Arabidopsis thaliana uncharacterized protein. (DOCX) [file pone.0182459.s001.docx]

| Gene | Gene Locus Tag | | ID In fame | |  | | Gene Description |  |
| --- | --- | --- | --- | --- | --- | --- | --- | --- |
| Pat6  ILP1  DUF  DJA  KRS  AP22  A1E  HSP40  RPS5  RPS11  2bBP19  2bBP78 | | At3G51670  At1G17210  At1G23580  At2G22360  At3G11710  At4G36980  At5G15140  [At1G80030](http://www.ncbi.nlm.nih.gov/gene/844343)  [At3G49080](http://www.ncbi.nlm.nih.gov/gene/824070)  At1G31817  At5G57460  At3G21295 | 824330  838292  838968  816768  820343  829852  831366  844343  824070  840071  835851  5008017 | C terminal 158aa  122aa in frame  lack C' 108aa  C terminal 214aa  full CDS  truncated 91 aa  lack N' 73aa  full CDS  C terminal 191aa  lack N' 48aa  C terminal 234 aa  C terminal 295aa | | cytosolic factor family protein / phosphoglyceride transfer family protein  IAP-like family protein 1  Arabidopsis thaliana hypothetical protein, uncharacterized protein mRNA  DNAJ heat shock family protein  lysyl-tRNA synthetase mRNA, lysyl-tRNA synthetase 1  Arabidopsis thaliana hypothetical protein, uncharacterized protein mRNA  mRNA for putative aldose 1-epimerase  molecular chaperone heat shock protein 40  30S ribosomal protein subunit S5 domain2-like superfamily protein  30S ribosomal protein subunit S11  Arabidopsis thaliana uncharacterized protein  Arabidopsis thaliana uncharacterized protein | | |
