# Supplementary figures and images for: The interaction between endogenous 30S ribosomal subunit protein S11 and Cucumber mosaic virus LS2b protein affects viral replication, infection and gene silencing suppressor activity
Source: PLoS One. 2017 Aug 14;12(8):e0182459. doi: 10.1371/journal.pone.0182459 (PMC5555695; doi:10.1371/journal.pone.0182459)

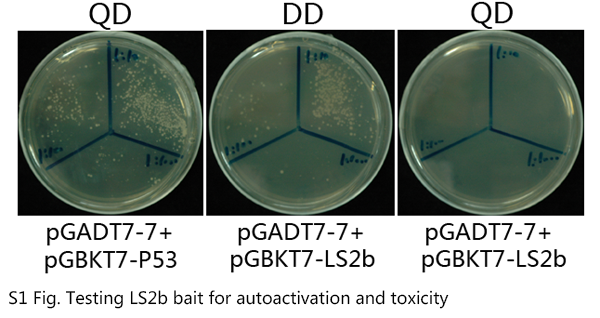

Supplement: S1 Fig — Confirmation of co-translation pGADT7-T with pGBKT7-P5 as positive control on QD medium plate. 1:10, 1:100,1:1000 gradient dilutions were spread on each one third of plates for illustration of co-translation ability. (TIF) [file pone.0182459.s002.tif]

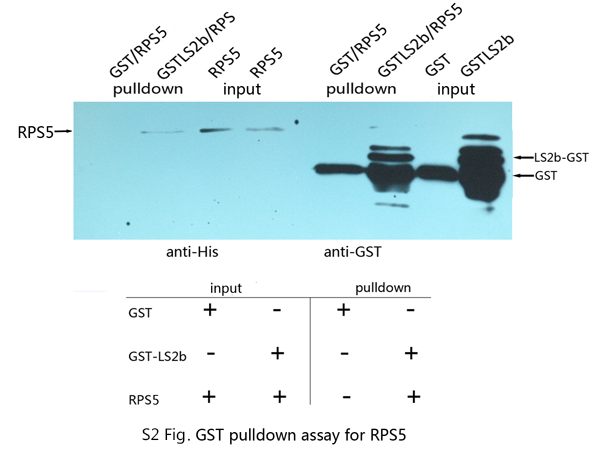

Supplement: S2 Fig — The presence of RPS5 was detected by immunoblot with anti-HIS antibody on left side of the membrane. The presence and expression of GST and GST-LS2b was confirmed by immunoblotting with anti-GST antibody on right side of the membrane. (TIF) [file pone.0182459.s003.tif]

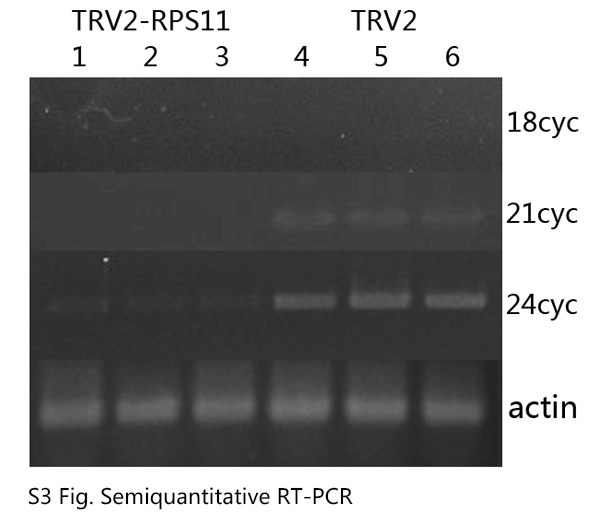

Supplement: S3 Fig — The upper, middle, lower panels of NbRPS11 mRNA were respectively PCR amplified products of 18th cycle, 21st cycle and 24th cycle. The mRNA of endogenous β-actin amplified at 24th cycle served as a loading control. (TIF) [file pone.0182459.s004.tif]
